# Losartan reduces ensuing chronic kidney disease and mortality after acute kidney injury

**DOI:** 10.1038/srep34265

**Published:** 2016-09-28

**Authors:** Shun-Yang Cheng, Yu-Hsiang Chou, Fang-Ling Liao, Chi-Chun Lin, Fan-Chi Chang, Chia-Hao Liu, Tao-Min Huang, Chun-Fu Lai, Yu-Feng Lin, Vin-Cent Wu, Tzong-Shinn Chu, Ming-Shiou Wu, Shuei-Liong Lin

**Affiliations:** 1 Graduate Institute of Physiology, College of Medicine, National Taiwan University, Taipei, Taiwan; 2 Renal Division, Department of Internal Medicine, National Taiwan University Hospital, Taipei, Taiwan; 3 Department of Internal Medicine, National Taiwan University Hospital Yun-Lin Branch, Yun-Lin, Taiwan; 4 Renal Division, Department of Internal Medicine, Taipei Medical University Hospital, Taipei, Taiwan

## Abstract

Acute kidney injury (AKI) is an important risk factor for incident chronic kidney disease (CKD). Clinical studies disclose that ensuing CKD progresses after functional recovery from AKI, but the underlying mechanisms remain illusive. Using a murine model representing AKI-CKD continuum, we show angiotensin II type 1a (AT1a) receptor signaling as one of the underlying mechanisms. Male adult CD-1 mice presented severe AKI with 20% mortality within 2 weeks after right nephrectomy and left renal ischemia-reperfusion injury. Despite functional recovery, focal tubular atrophy, interstitial cell infiltration and fibrosis, upregulation of genes encoding angiotensinogen and AT1a receptor were shown in kidneys 4 weeks after AKI. Thereafter mice manifested increase of blood pressure, albuminuria and azotemia progressively. Drinking water with or without losartan or hydralazine was administered to mice from 4 weeks after AKI. Increase of mortality, blood pressure, albuminuria, azotemia and kidney fibrosis was noted in mice with vehicle administration during the 5-month experimental period. On the contrary, these parameters in mice with losartan administration were reduced to the levels shown in control group. Hydralazine did not provide similar beneficial effect though blood pressure was controlled. These findings demonstrate that losartan can reduce ensuing CKD and mortality after functional recovery from AKI.

Acute kidney injury (AKI) is responsible for approximately 2 million deaths annually worldwide^1^,^2^. Numerous studies have demonstrated that AKI is associated with increases of hospital duration and expenditure, risk for infection and mortality^1^,^2^,^3^,^4^,^5^,^6^,^7^,^8^,^9^,^10^,^11^. Patients requiring dialysis therapy for postoperative AKI have mortality rates ranging from 40 to 80%, similar to that seen in patients with postoperative cardiac arrest^1^,^2^,^3^,^4^,^5^,^6^,^7^,^8^,^9^,^10^,^11^. Even though the disease severity without need for dialysis therapy, AKI represented by small and reversible changes in serum levels of creatinine is increasingly recognized as a risk for mortality in patients undergoing cardiac surgery^11^,^12^,^13^,^14^.

AKI patients that can be supported through the episode have a good chance of functional recovery because of the kidney’s capacity for repair. The cellular hallmark of kidney repair is a rapid proliferative response ultimately leading to the restoration of structure and function of the nephrons. The origin of the cells that replace the injured tubular epithelia has been in debate for decades until several recent lines of evidence suggesting an intrarenal source^15^,^16^,^17^. Compatible with these findings, bone marrow-derived stem cells were shown not to regenerate the injured tubular epithelia directly^15^,^18^. One of the fates of the recruited bone marrow-derived stem cells are macrophages who promote repair of the injured tubular epithelial cells through synthesizing WNT 7b and the other growth factors^19^,^20^,^21^.

There is substantial progress in the field of AKI over the past 10 years^6^,^7^,^8^,^9^,^10^,^11^. The previous conventional wisdom that AKI survivors with fully recovered renal function tend to do well appears to be flawed^14^,^22^,^23^,^24^,^25^,^26^,^27^,^28^,^29^,^30^,^31^,^32^,^33^,^34^. In agreement with the other independent studies^28^,^29^,^30^,^31^,^32^,^33^, not only the risks for cardiovascular events and long-term mortality but also the ensuing chronic kidney disease (CKD) and end-stage renal disease (ESRD) increased substantially after discharge from hospital in our cohort of dialysis-requiring AKI patients^24^,^25^. Moreover, we demonstrated a steady decline of renal function and an increased risk for long-term mortality after discharge in non-dialysis-requiring AKI patients^14^. Similar conclusions have been made in the other independent studies^22^,^23^,^32^. It is hence possible that pathological process remains ongoing in the repairing kidney and underlies the mechanisms for the ensuing CKD even though the clinical parameters of renal function are not apparently abnormal in patients recovering from AKI^34^,^35^,^36^,^37^,^38^,^39^.

There are several plausible mechanisms by which patients experiencing AKI may develop ensuing CKD, including hypertension, hyperfiltration, glomerulosclerosis, interstitial fibrosis, persistent inflammation, tubular epithelial cell cycle arrest and tubular shortening^31^,^34^,^35^,^36^,^37^,^38^,^39^,^40^. It is noteworthy that expression of *Havcr1* and *Lcn2*, which encode kidney injury molecule-1 (KIM-1) and neutrophil gelatinase-associated lipocalin (NGAL) respectively, is highly upregulated in the late stage of repaired kidneys after ischemia-reperfusion injury (IRI)^35^. Furthermore, renal tubular regeneration occurs in most models of experimental AKI, but the recovery is often incomplete depending on the severity of injury^37^,^38^. Kidneys with focal areas that do not repair fully will present tubular decomposition and nephron loss^38^,^39^,^40^.

Reduction in renal mass and nephron number appears to be an important determinant of the ensuing CKD in AKI patients, however the molecular mechanisms underlying the AKI-CKD transition remain illusive. This study was therefore conducted to establish a murine model of AKI-CKD continuum and then delineate the mechanisms underlying the ensuing CKD progression after functional recovery from AKI.

## Results

### Establish a murine model of severe AKI with ensuing CKD after functional recovery

**Figure 1:**
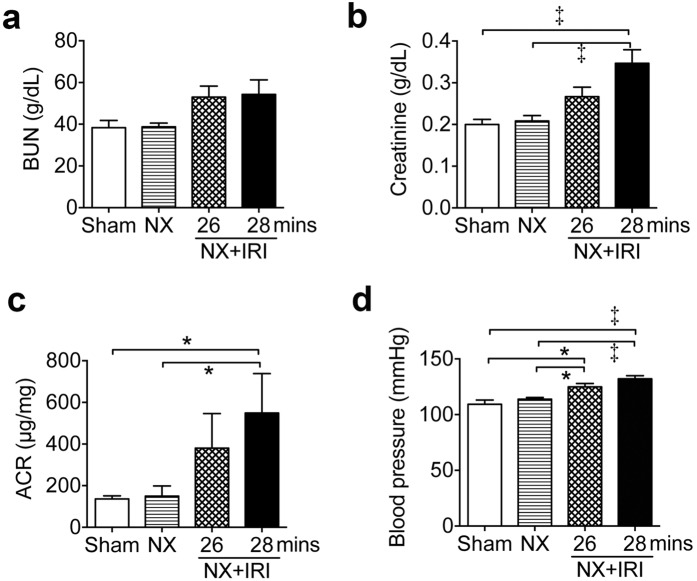
AKI induced by nephrectomy followed by contralateral renal ischemia-reperfusion injury leading to development of ensuing CKD. (**a**,**b**) Blood urea nitrogen (BUN) and creatinine plasma levels, (**c**) urine albumin-creatinine ratio (ACR), and (**d**) systolic blood pressure (BP) in adult male mice 3 months after sham operation, nephrectomy (NX) and NX followed by 26- or 28-minute ischemia/reperfusion to contralateral kidney (NX+IRI-26 min, NX+IRI-28 min) respectively. *N* = 10, 20, 18 and 16 for sham, NX, NX+IRI-26 min, and NX+IRI-28 min respectively. *P < 0.05, ^‡^*P* < 0.001.

**Figure 2:**
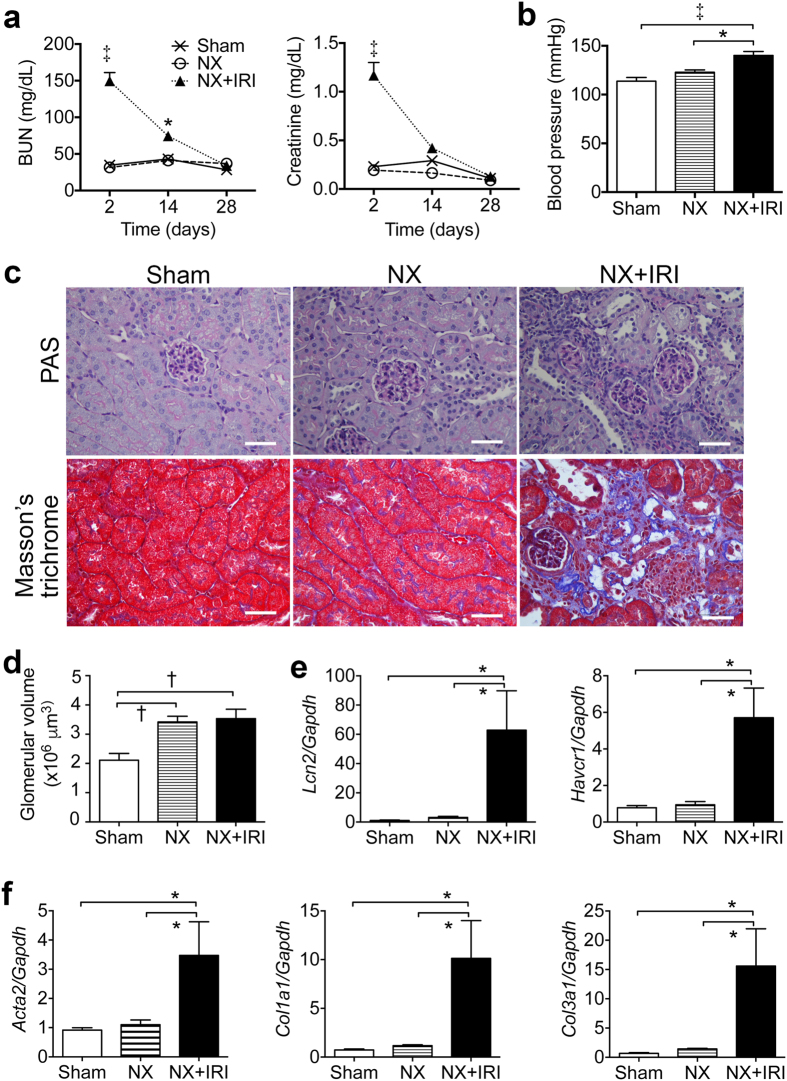
A murine AKI model shows abnormal renal pathology and ongoing injury after functional recovery. (**a**) BUN and creatinine plasma levels in adult male mice subjected to sham operation, NX and NX+IRI. **(b**) Systolic blood pressure at day 28. **(c**) Representative images of periodic acid-Schiff (PAS) and Masson’s trichrome stain of kidneys at day 28. Scale bar, 100 μm. **(d**) Renal glomerular volume at day 28. **(e**,**f**) Quantitative polymerase chain reaction (QPCR) for gene expression in kidneys at day 28. AKI marker genes *Lcn-2* and *Havcr-1* encoded neutrophil gelatinase-associated lipocalin and kidney injury molecule-1 respectively. Pro-fibrotic genes *Acta2*, *Col1a1* and *Col3a1* encoded α-smooth muscle actin, collagen I α1 and Collagen III α1 chains respectively. The expression levels were normalized by glyceraldehyde *3-phosphate dehydrogenase (Gapdh*). *P < 0.05, ^†^*P* < 0.01, ^‡^*P* < 0.001. *N* = 6 for each group of sham and NX in each time point; *N* = 13, 11 and 11 for NX+IRI group at day 2, 14 and 28 respectively.

**Figure 3:**
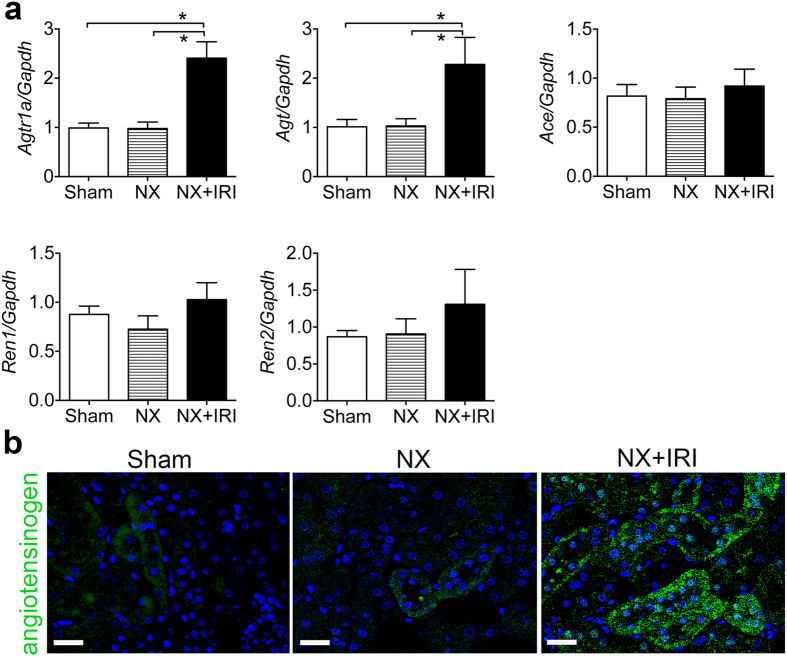
Activation of renin-angiotensin system (RAS) in repaired kidney after functional recovery from AKI. **(a**) QPCR for RAS gene expression in kidneys at day 28 after AKI. *Agtr1a*, *Agt*, *Ace*, *Ren1* and *Ren2* encoded angiotensin II type 1a (AT1a) receptor, angiotensinogen, angiotensin converting enzyme, *Renin 1* and *Renin 2* respectively. *P < 0.05. *N* = 6 for each group of sham and NX; *N* = 11 for NX+IRI group. **(b**) Representative confocal images of angiotensinogen staining in kidneys at day 28. Scale bar, 20 μm.

In the first, we tried to establish a murine model to test the hypothesis that an episode of severe AKI would lead to ensuing CKD after functional recovery. AKI induced by IRI in adult male CD1 mice was used for this experiment. Because one of the kidneys with more injury would show scaring and atrophy by 4 weeks after injury even though the other kidney appeared hypertrophic in the mouse undergoing bilateral renal IRI, we induced AKI by right nephrectomy (NX) first and then left renal IRI at 8 and 10 weeks of age respectively. Because clinical studies disclose that the severer AKI is, the more likely CKD develops after functional recovery, we subjected the mice to renal IRI with different duration of warm ischemia. The mortality rates in mice subjected to 26-, 28- and 30-minute ischemia of left kidney 2 weeks after right NX were 10%, 20% and 60% respectively within 2 weeks after AKI. In contrast, all mice in the control groups of sham surgery or NX survived. We therefore did not analyze NX+IRI-30 min further. Plasma levels of blood urea nitrogen (BUN) and creatinine were not different between groups of sham, NX, NX+IRI-26 min and NX+IRI-28 min 2 weeks after surgery, suggesting functional recovery from AKI in groups of NX+IRI. To study whether the model would manifest abnormal renal function again in later time point, we extended the observation and found that BUN and creatinine plasma levels increased and significantly higher in NX+IRI-28 min group 3 months after surgery (Fig. 1a,b). Urine albumin-creatinine ratio (ACR) and systolic blood pressure (BP) increased in NX+IRI-28 min group, too (Fig. 1c,d). We therefore chose 28-minute ischemia and then reperfusion to the left kidney 2 weeks after right NX as our AKI model that presented notable severity but did not lead to too high mortality rate to analyze the ensuing disease process after functional recovery. The plasma levels of BUN and creatinine 2 weeks after NX were stable and not different from those of sham-operated mice (sham *versus* NX, 0.292 ± 0.067 *versus* 0.164 ± 0.061 mg/dL). Day 2 plasma levels of BUN and creatinine after IRI were 150.11 and 1.21 mg/dL respectively in mice subjected to NX+IRI, which were significantly higher than those of sham and NX mice (Fig. 2a). The azotemia in NX+IRI group recovered to the comparable levels shown in sham and NX mice by 4 weeks after IRI (Fig. 2a). However, BP of NX+IRI group was notably higher than that of control groups (Fig. 2b). Periodic acid-Schiff (PAS) staining revealed significant increase of renal glomerular volume in both NX and NX+IRI groups, whereas focal interstitial fibrosis, cell infiltration and tubular atrophy were only shown in kidneys of NX+IRI groups (Fig. 2c,d). AKI marker genes *Lcn-2* and *Havcr-1* which encoded neutrophil gelatinase-associated lipocalin (NGAL) and kidney injury molecule-1 (KIM-1) respectively continued to be upregulated in kidneys of NX+IRI group even 4 weeks after surgery (Fig. 2e). Upregulation of pro-fibrotic genes *Acta2*, *Col1a1* and *Col3a1*, encoding α-smooth muscle actin, collagen I α1 and collagen III α1 chains respectively, was found in kidneys of NX+IRI group, too (Fig. 2f). Further analyses identified upregulation of genes *Agt* and *Agtr1a* that encoded angiotensinogen and angiotensin II type 1a (AT1a) receptor respectively in kidneys of NX+IRI group (Fig. 3a), suggesting the activation of intrarenal renin-angiotensin system (RAS). Angiotensinogen was weakly detectable in focal tubular epithelial cells of kidneys of sham and NX mice by immunofluorescence, but the expression was increased robustly after NX+IRI (Fig. 3b).

**Figure 4:**
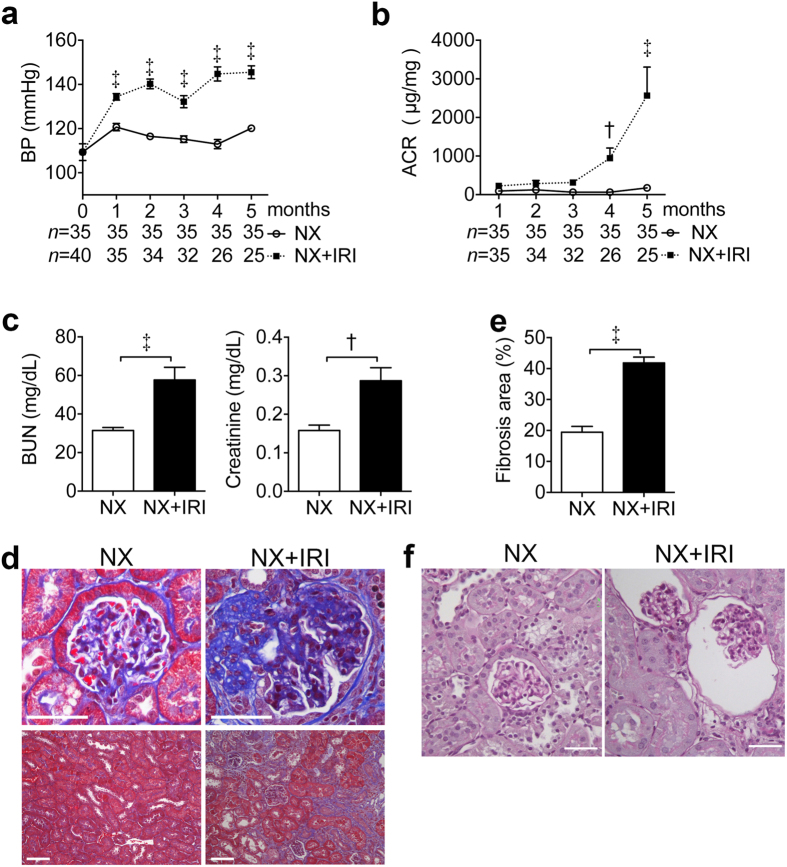
CKD development after AKI in a larger mouse cohort. (**a**,**b**) Time course systolic BP and urine ACR after NX or NX+IRI. **(c**) Plasma levels of BUN and creatinine at 5 months after surgery. **(d**) Representative images of Masson’s trichrome stain in kidneys at 5 months after surgery. **(e)** Quantification of interstitial fibrosis area in Masson’s trichrome stain of kidneys. **(f**) Representative images of PAS stain of kidneys showing shrunken glomerular tuft with widened Bowman’s space at 5 months after surgery. Scale bar, 100 μm. ^†^*P* < 0.01, ^‡^*P* < 0.001. *N* was shown in (**a**,**b**).

We then extended the observation to 5 months in a larger mouse cohort after NX or NX+IRI. The elevation of BP was noted since 1 month after NX+IRI and persisted thereafter (Fig. 4a). Urine ACR increased gradually after NX+IRI (Fig. 4b). Plasma levels of BUN and creatinine were also higher in NX+IRI group 5 months after surgery (Fig. 4c). Histological examination revealed remarkable nephrosclerosis and interstitial fibrosis in NX+IRI mice, but not in NX mice (Fig. 4d,e). Moreover, shrunken tufts with widened Bowman’s space suggesting glomerular ischemia were only shown in 8.62% of glomeruli in the kidneys of NX+IRI mice (Fig. 4f).

These data supported that NX+IRI could be the model for studying ensuing CKD after functional recovery from AKI.

### Losartan reduces mortality and ensuing CKD after severe AKI

**Figure 5:**
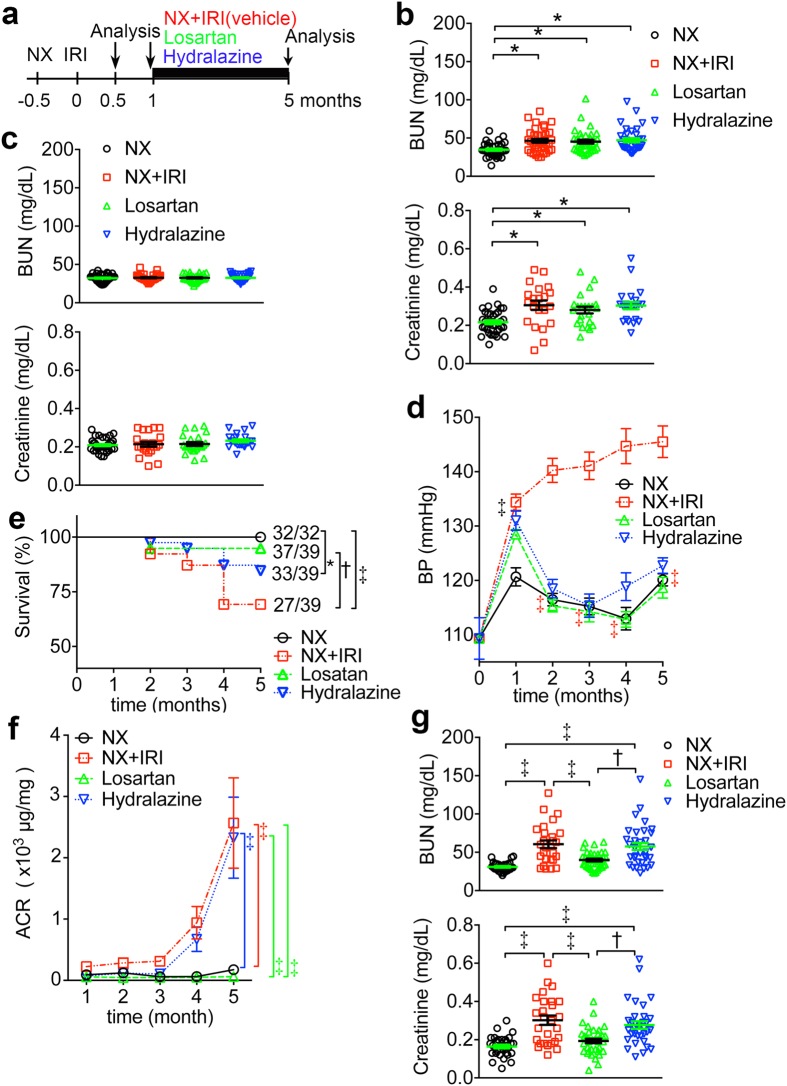
Losartan reduces mortality and ensuing CKD after severe AKI. (**a**) Plasma BUN and creatinine were analyzed 14 and 27 days after IRI or sham surgery and then the mice were administered water vehicle, losartan, or hydralazine since 1 month after NX+IRI. NX only mice served as the sham control. All mice were sacrificed for analyses 5 months after surgery. *N* for each group at each time point was shown in (**e**). (**b**) Plasma levels of BUN and creatinine 14 days after surgery. (**c**) Plasma levels of BUN and creatinine 27 days after surgery. *N* = 32 in NX group, and 39 for each group of NX+IRI in (**b**,**c**). Each symbol represents the data from each mouse. **(d**) Time course systolic BP. NX *versus* other 3 groups at 1 month; NX+IRI *versus* other 3 groups at 2, 3, 4, and 5 month. **(e**) The percentage of survival. **(f**) Time course urine ACR. **(g**) Plasma levels of BUN and creatinine at 5 month when *N* = 32 (NX), 27 (NX+IRI), 37 (Losartan) and 33 (Hydralazine) as shown in (**e**). *P < 0.05, ^†^*P* < 0.01, ^‡^*P* < 0.001.

To get insight into the role of BP elevation and intrarenal RAS activation in the ensuing CKD and disease progression after functional recovery from AKI, mice were administered a direct-acting vascular smooth muscle relaxant hydralazine (Hydralazine group), an AT1a receptor antagonist losartan (Losartan group) or water vehicle control (NX+IRI group as the AKI-CKD control) since 1 month after NX+IRI. NX only mice (NX group) served as the sham control (Fig. 5a). Plasma levels of BUN and creatinine were obtained 2 weeks after IRI or sham surgery and then the mice were randomized to receive specific treatments from 1 month (Fig. 5b). The levels of BUN and creatinine in all NX+IRI groups were not different from those in NX groups one day before starting specific treatments (Fig. 5c). BP was elevated but not different in NX+IRI mice before starting specific treatment (Fig. 5d). Administration of losartan or hydralazine to NX+IRI mice reduced BP to the levels obtained in NX group (Fig. 5d). However, BP elevation persisted in NX+IRI group (Fig. 5d). All mice in NX group survived to 5 months after surgery, but some mice in NX+IRI group were found dead in this period (Fig. 5e). Very high plasma levels of BUN and creatinine were verified before death in some mice. The survival rates of NX and NX+IRI groups were 32/32 (100%) and 27/39 (69.2%) respectively 5 months after surgery (Fig. 5e, *P* < 0.001). Administration of losartan, not hydralazine, improved the survival of NX+IRI mice significantly (Fig. 5e, Losartan *versus* NX+IRI, 37/39 *versus* 27/39, *P* < 0.01). Hydralazine did not improve survival of mice after NX+IRI (Fig. 5e, Hydralazine *versus* NX+IRI, 33/39 *versus* 27/39, *P* = 0.1027), and the survival of mice in Hydralazine group was still worse than in NX group (Fig. 5e, Hydralazine *versus* NX, 33/39 *versus* 32/32, *P* < 0.05). The following analyses were therefore derived from the survived mice only. The anti-hypertensive benefit of hydralazine did not improve urine ACR, but on the contrary, urine ACR did not increase at all in mice of Losartan group (Fig. 5f). At endpoint of this study 5 months after surgery, losartan administration prevented the re-elevation of plasma BUN and creatinine in mice after functional recovery from NX+IRI, but hydralazine did not (Fig. 5g).

### Losartan attenuates kidney fibrosis after severe AKI

**Figure 6:**
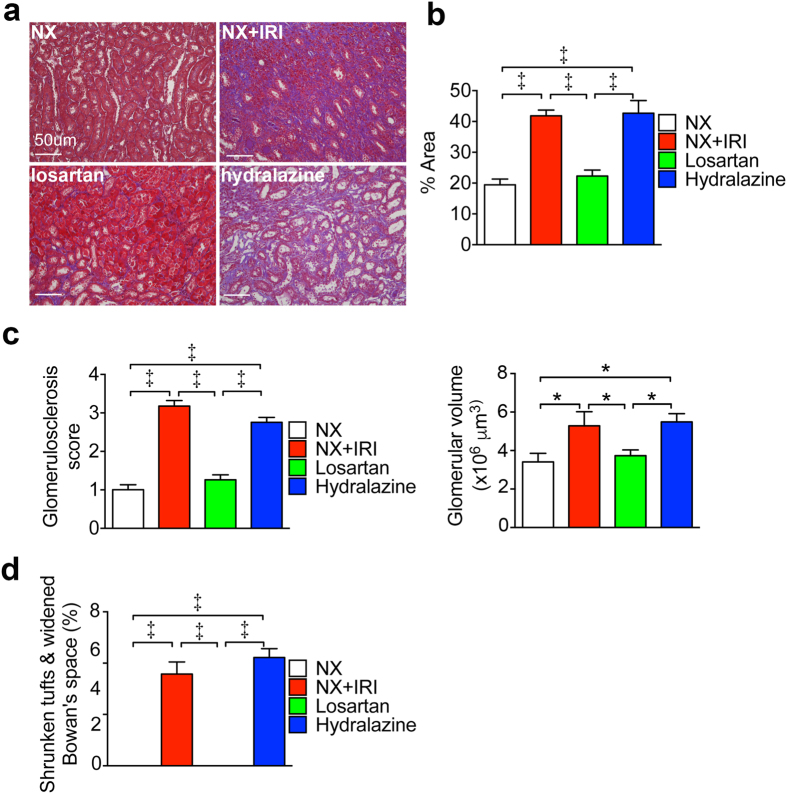
Losartan attenuates fibrosis in ensuing CKD after AKI. **(a**,**b**) Representative images and quantification of interstitial fibrosis area in kidneys by Masson’s trichrome stain at 5 months after surgery and specific treatment when *N* = 32 (NX), 27 (NX+IRI), 37 (Losartan) and 33 (Hydralazine) as shown in Fig. 5e. (**c**,**d**) Renal glomerular volume, glomerulosclerosis and the percentage of glomeruli with shrunken tufts and widened Bowman’s space. *P < 0.05, ^‡^*P* < 0.001.

**Figure 7:**
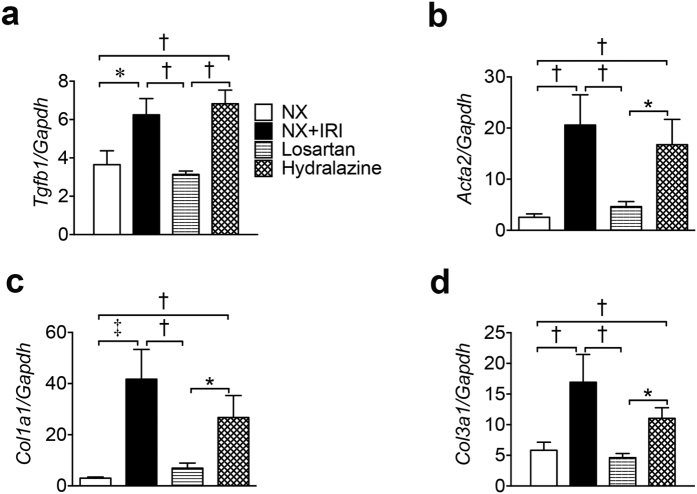
Losartan attenuates pro-fibrotic genes in kidney after AKI. QPCR of renal pro-fibrotic genes (**a**) *transforming growth factor-b1 (Tgfb1*), **(b**) *Acta2*, **(c**) *Col1a1,* and **(d**) *Col3a1* at 5 months. *N* = 32 (NX), 27 (NX+IRI), 37 (Losartan) and 33 (Hydralazine) as shown in Fig. 5e. *P < 0.05, ^†^*P* < 0.01, ^‡^*P* < 0.001.

To clarify the mechanisms underlying the preventive effect of losartan on CKD progression after functional recovery from AKI, we studied renal pathology and gene expression. Marked deposition of extracellular matrix in kidneys of NX+IRI and Hydralazine groups was shown in Masson’s trichrome stain, which was almost completely attenuated in kidneys of Losartan group (Fig. 6a,b). Moreover, the increases of glomerular volume and glomerulosclerosis in NX+IRI mice were also attenuated in mice of Losartan group (Fig. 6c). The percentage of glomeruli with shrunken tufts and widened Bowman’s space in the kidneys of NX+IRI mice was prevented by losartan treatment, too (Fig. 6d). In line with the findings in renal pathology, the upregulation of *Tgfb1* (encoding transforming growth factor-β1), *Acta2*, *Col3a1* and *Col1a1* in kidneys of NX+IRI mice was attenuated by administration of losartan (Fig. 7a–d). These protective effects were not seen in Hydralazine group (Figs 6 and 7).

## Discussion

The beneficial effect of RAS blockade in diabetic kidney disease and proteinuric non-diabetic kidney disease is well known^41^. RAS blockade with ACE inhibitors or AT1a receptor antagonists have been widely applied to protect CKD from progression clinically^41^,^42^. One of the most important mechanisms for intrarenal RAS activation is the reduction of nephron number^36^,^43^. RAS activation is good for maintaining glomerular filtration and compensating the function loss, however vicious cycle leading to glomerulosclerosis, interstitial fibrosis and further reduction of nephron number will result in CKD progression^36^,^43^. RAS blockade is usually avoided during the acute phase of AKI patients, but the role of RAS activity in kidney injury is not clear indeed^44^,^45^,^46^. Although extensive studies in animal AKI models have been made, no specific therapy that benefits the human AKI, either in injury attenuation, recovery promotion or ensuing CKD prevention has been discovered^47^. Our cohort study found the association of pre-operative RAS blockade with a reduction of AKI after elective cardiac surgery^44^. A recent rat study showed that RAS blockade 3 days before bilateral renal IRI effectively prevents ensuing CKD despite no effect on initial AKI severity^45^. It is not clear whether RAS blockade before AKI produces ischemic preconditioning effect thereby preventing ensuing CKD^48^. In this study the murine model of NX+IRI provided a reliable tool to understand the renal pathology and molecular markers that existed in the repairing kidneys after functional recovery from severe AKI and emerged as potential targets to prevent CKD. Our findings support that pathological process, including focal tubular atrophy, interstitial inflammation and fibrosis, remained in the repairing kidneys even though azotemia returned to normal range by 4 weeks after AKI. The markers for tubular injury and inflammation including *Lcn-2* and *Havcr-1* encoding NGAL and KIM-1 respectively continued to be highly expressed in the repairing kidneys, suggesting the presence of ongoing injury in kidneys despite no azotemia. Although albuminuria was not evident in mice 4 weeks after NX+IRI, elevation of BP was significant, a finding in line with a recent study which showed elevated BP in the survivors of AKI in a large cohort^31^. In addition to the abovementioned abnormal pathology and injury markers, upregulated expression of *Agt* and *Atr1a* encoding angiotensinogen and AT1a receptor respectively suggested intrarenal RAS activation in repairing kidneys after AKI. Systemic RAS activation could not be clarified in this study due to technical issue in measuring plasma renin activity, angiotensin and aldosterone of mice in our laboratory. Critical reduction of nephron number may be the underlying mechanism for intrarenal RAS activation to maintain glomerular filtration by residual nephrons after AKI. Kidneys repairing from acute injury were found to retain abnormal pathology and ongoing injury. Although plasma biochemical parameters showed recovery of renal function, abnormal pathology, ongoing injury and RAS activation in repairing kidneys might lead to vicious cycle shown in remnant nephropathy^36^,^49^.

Our experiments disclosed that repair and regeneration in injured kidneys were not complete, findings in line with previous reports by independent groups^35^,^37^,^38^. In addition to the highly upregulated *Havcr1* and *Lcn2* late after AKI, increased expression of genes encoding amiloride binding protein-1, vascular cell adhesion molecule, and endothelin has been shown in the repaired kidneys, which is believed to explain the salt-sensitive hypertension after AKI and contribute to the CKD progression^31^,^35^,^50^,^51^. In AKI induced by aristolochic acid, severe bilateral IRI, unilateral IRI or unilateral ureteral obstruction, cell cycle G2/M-arrested proximal tubular epithelial cells have been shown to activate pro-fibrotic signaling for progressive renal fibrosis^37^,^52^. Compared to milder bilateral IRI, severe bilateral IRI leads to residual elevation of serum creatinine and the presence of interstitial fibrosis in focal area of kidneys by 6 weeks after injury^37^. These findings support the clinical observation that severe AKI may result in progressive CKD or ESRD^28^,^29^,^30^. Our murine model simulating severe AKI followed by normalization of azotemia, then development of progressive CKD and death further advance our knowledge in the AKI-CKD continuum. Since renal tubules have a limited capacity to repair, AKI results in significant shortening of renal tubules associated with atubular glomeruli, loss of nephrons, and interstitial fibrosis^38^. Our results further supported that persistent abnormalities including focal tubular atrophy, interstitial inflammation and fibrosis were ongoing even though BUN and creatinine plasma levels returned to normal range, and the ongoing pathological abnormalities would lead to more and more glomerulosclerosis, tubular atrophy and interstitial fibrosis.

The murine model of AKI-CKD continuum we used in this study simulated the most popular AKI of patients due to shock followed by resuscitation, and complication after cardiopulmonary bypass, etc. Although the progression of ensuing CKD was quite similar to that of 5/6 subtotal NX model, our model showed quick elevation of plasma BUN and creatinine at day 2 after IRI, normalization thereafter, and then elevation again accompanied with elevated BP and urine ACR, which was different from progressive azotemia shown in 5/6 subtotal NX model^49^,^53^. Different from our aim to study the effect of AKI severity on ensuing CKD and the molecular mechanism underlying the disease progression, Polichnowski and colleagues have performed IRI in rats 2 weeks after 75% renal mass reduction (RMR), 50% RMR, or sham operation to determine the impact of preexisting CKD on the severity of and recovery from AKI^39^. Their findings support that preexisting severe (75%) RMR will compromise tubular repair, diminish microvascular density and promote fibrosis after IRI, thereby serum creatinine remaining elevated during late recovery.

More severe AKI is clinically associated with an increase in the odds of developing elevated BP after functional recovery^31^. However, our data did not support BP lowering therapy by direct vasodilator hydralazine as an effective strategy for prevention of ensuing CKD and ESRD after AKI. In contrast, RAS blockade with AT1a receptor antagonist losartan after functional recovery from AKI represented an effective strategy to reduce the ensuing CKD and mortality. Future studies are needed to explore the protective effect of RAS blockade in clinical patients.

In conclusion, mice after 2-step surgery including NX followed by IRI of contralateral kidney represent a good model for studying AKI-CKD continuum. Our data support ongoing injury and RAS activation in the repairing kidneys after AKI. RAS blockade with losartan is effective to reduce ensuing CKD and mortality.

## Methods

### Animal model of AKI

Adult (8–12 weeks) male CD1 mice were anesthetized with ketamine/xylazine (100/10 mg/kg, intraperitoneally) and subjected to right NX. Two weeks later, left kidney was clamped with a non-traumatic micro-aneurysm clip to perform IRI under the homeothermic blanket system (Stoelting Co. Wood Dale, IL) which contained a rectal thermal probe and a heating pad to maintain the core body temperature at 37 ^o^C. Right NX only was performed in control mice. All of the animal experiments including any relevant details were performed in accordance with relevant guidelines/regulations and approved by the institutional committee at National Taiwan University College of Medicine.

### Experimental groups

NX+IRI mice were divided into 3 groups receiving vehicle (drinking water only), losartan potassium (0.1 g/L in the drinking water; Sigma, St. Louis, MO), or hydralazine hydrochloride (0.06 g/L in the drinking water; Sigma) from one month after IRI surgery. Age-matched NX mice served as the control.

### Tissue preparation and histology

Mouse tissues were prepared and stained as previously described^54^. Primary antibody against angiotensinogen (#28101, Immuno-Biological Laboratories Co., Gunma, Japan) was used for immunolabeling. Fluorescence-conjugated secondary antibody labeling (Jackson Immunoresearch Laboratories, West Grove, PA), 4′,6-diamidino-2-phenylindole (DAPI) staining, Vectashield mounting, image capture and processing were carried out as previously described^54^. Interstitial fibrosis was quantified using Fovea Pro 4.0 in pictures of Masson’s trichrome stained sections taken at magnification X200 (Reindeer Graphics, Asheville, NC). To determine the glomerular volume, PAS stained pictures of at least 15 intact glomeruli with a vascular pole and no less than 20 nuclei were taken at magnification X400. The mean glomerular random cross-sectional area (A_G_) was measured using Fovea Pro 4.0. The glomerular volume was then calculated as by (b/k)6(A_G_)^3/2^, where b = 1.38 was the shape coefficient for spheres and k = 1.1 was the size distribution coefficient^55^. Glomerulosclerosis was defined by the presence of PAS stain +  material within the glomeruli using a semiquantitative score. To determine the average glomerulosclerosis score for each kidney, pictures of at least 15 intact glomeruli with a vascular pole and no less than 20 nuclei were taken at magnification X400. Sclerosis score for each glomerulus was graded from 0 to 4 (0, no changes; 1, changes affecting  < 25% of the glomeruli; 2, changes affecting 25 to 50%; 3, changes affecting 50 to 75%; 4, changes affecting 75 to 100%).

### Quantitative polymerase chain reaction (QPCR)

Total RNA was extracted using RNeasy Mini Kit (Qiagen). Purity was determined by A260 to A280. cDNA was synthesized using iScript cDNA Synthesis Kit (Bio-Rad Laboratories, Hercules, CA). QPCR was performed using methods described previously^54^. The expression levels were normalized by glyceraldehyde *3-phosphate dehydrogenase (Gapdh*). The specific primer pairs used for QPCR are listed in supplementary Table 1.

### Blood pressure analysis in mice

Systolic blood pressures were measured in conscious mice using a computerized tail-cuff method (BP-2000; Visitech Systems, Apex, NC) described previously^49^. Mice were acclimatized to the system before the initiation of studies. Systolic blood pressures were measured the day before surgery or euthanasia. Blood pressures were measured for five times to determine the mean levels.

### Biochemical analyses of mouse plasma and urine

Plasma and urine were collected and kept frozen in aliquots until analyses performed in Laboratory Animal Center, National Taiwan University College of Medicine. Urine albumin-creatinine ratio was calculated by dividing the spot urine albumin-creatinine concentration.

### Statistics

Data were expressed as mean ± standard error (SE), and analyzed using GraphPad Prizm (GraphPad Software, La Jolla, CA). Comparisons between data with two factors were performed using two-way ANOVA with post hoc analysis for multiple group comparisons using Tukey’s method. Comparisons between data with one factor but two groups were performed using student’s T-test. Data with one factor and three or more groups were analyzed by one-way ANOVA with post hoc analysis for multiple group comparisons using Tukey’s method. Survival analysis was performed using the Mantel-Cox method. *P* < 0.05 was considered significant.

## Additional Information

**How to cite this article**: Cheng, S.-Y. *et al.* Losartan reduces ensuing chronic kidney disease and mortality after acute kidney injury. *Sci. Rep.* **6**, 34265; doi: 10.1038/srep34265 (2016).

## Supplementary Material

Supplementary Information
