# Supplementary material for: Losartan reduces ensuing chronic kidney disease and mortality after acute kidney injury
Source: Sci Rep. 2016 Sep 28;6:34265. doi: 10.1038/srep34265 (PMC5039710; doi:10.1038/srep34265)
Supplement: Supplementary Information [file srep34265-s1.pdf]

Cheng et al. Supplementary information

Losartan reduces ensuing chronic kidney disease and mortality after acute kidney injury

Shun-Yang Cheng, Yu-Hsiang Chou, Fang-Ling Liao, Chi-Chun Lin, Fan-Chi Chang, Chia-Hao Liu, Tao-Min Huang, Chun-Fu Lai, Yu-Feng Lin, Vin-Cent Wu, Tzong-Shinn–Chu, Ming-Shiou Wu, Shuei-Liong Lin

Supplementary Table 1. Primer sequences used in QPCR

Target	Primer	Sequence (5' to 3')
Ace	Forward	TGCCCCTGGAACCTGATCTA
	Reverse	TGTAGCCATTGAGCTTGGCA
Acta2	Forward	CTGACAGAGGCACCACTGAA
	Reverse	CATCTCCAGAGTCCAGCACA
Agt	Forward	AGGTTGGCGCTGAAGGATAC
	Reverse	GATGTATACGCGGTCCCCAG
Agtr1a	Forward	AGTTGGGAGGGACTGGATGA
	Reverse	GTTAAGTCCGGGAGAGCAGC
Col1a1	Forward	GAGCGGAGAGTACTGGATCG
	Reverse	GTTCGGGCTGATGTACCAGT
Col3a1	Forward	ACCAAAAGGTGATGCTGGAC
	Reverse	GACCTCGTGCTCCAGTTAGC
Gapdh	Forward	ACGGCCGCATCTTCTTGTGCA
	Reverse	AATGGCAGCCCTGGTGACCA
Havcr1	Forward	AAACCAGAGATTCCCACACG
	Reverse	GTCGTGGGTCTTCCTGTAGC
Lcn2	Forward	ATGCACAGGTATCCTCAGGT
	Reverse	TGGCGAACTGGTTGTAGTCC
Ren1	Forward	GCACCGCTACCTTTGAACGA
	Reverse	TCGCCGTAGTACTGGGTATTC
Ren2	Forward	TCTCTGGGCACTCTTGTTGC
	Reverse	CCACGGGGGAGATAAGATCAG

Tgfb1	Forward	GGACTCTCCACCTGCAAGAC
	Reverse	GACTGGCGAGCCTTAGTTTG
